# Unveiling aromas: Virtual reality and scent identification for sensory analysis

**DOI:** 10.1016/j.crfs.2024.100698

**Published:** 2024-02-15

**Authors:** Abdul Hannan Bin Zulkarnain, Dalma Radványi, Dorina Szakál, Zoltán Kókai, Attila Gere

**Affiliations:** a Institute of Food Science and Technology, Hungarian University of Agriculture and Life Sciences, H-1118, Budapest, Villányi út. 29-31, Hungary; b Department of Hospitality, Faculty of Commerce, Hospitality and Tourism, Budapest Business University, H-1045, Budapest, Alkotmány utca 9-11., Hungary; c Institute of Agribusiness, Hungarian University of Agriculture and Life Sciences, H-1118, Budapest, Villányi út. 29-31, Hungary

**Keywords:** Aroma, Multiple factor analysis, Virtual sensory laboratory, Smelling test, Visual

## Abstract

Sensory analysis is crucial for optimizing experiences in various fields, including food, cosmetics, and product design. Traditional methods can be inefficient and imprecise. This study introduces a novel approach by blending Virtual Reality (VR) technology with scent identification techniques. The aim is to investigate whether the visual representation of food products affects scent perception. Limited research has explored the use of VR in scent identification, which is especially relevant when altering the food environment setting. A virtual sensory laboratory was developed to mimic MATE's sensory booth. Sixty participants, all MATE students, were involved in this study. This method offers a potential means to streamline scent identification and reduce human bias in sensory analysis. In summary, the combination of VR technology and scent identification presents a fresh methodological approach to sensory analysis, where both scent and exposure are influenced by the environment or imagery. This concept delves into cross-modal correspondences and the role of sensory cues in shaping our perception of food odours within the VR setting.

## Highlights

• The image or the surroundings affect scent and exposure.
• Odour recognition tests can generally be conducted in virtual reality environments.
• In virtual reality environments, scent can significantly affect presence, perception, and user experience.

## Graphical abstract

**Figure:**
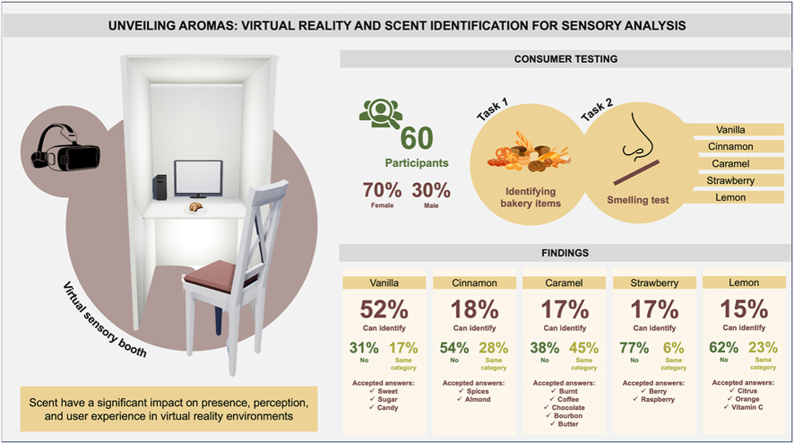

## 1 Introduction

Virtual reality (VR) has emerged as a promising tool for sensory analysis in the field of food science. The use of VR technology allows for the creation of immersive environments that can enhance the sensory testing of foods (Ammann et al., 2020). By providing artificially applied context, such as visual and auditory cues, VR can simulate real-world scenarios and elicit more realistic sensory responses from participants (Gere et al., 2021; Fuentes et al., 2021). This has significant implications for the food industry, as it offers new opportunities for understanding consumer sensory perceptions and designing products that meet their preferences and expectations (Crofton et al., 2019).

One area of research in VR sensory analysis is the evaluation of scents and their impact on cognitive processes. A study conducted by Mancini et al. (2021) investigated the effects of lemon and lavender scents on cognitive resources during a train journey in VR (Mancini et al., 2021). The findings showed that the lavender scent led to a higher demand for cognitive resources compared to the lemon scent. However, there were no differences in self-reported pleasantness and involvement between the two scents. This study demonstrates the potential of using scents in VR to influence cognitive experiences and highlights the importance of considering scent as a factor in sensory analysis. Olfactory perception and presence in a VR food environment have also been explored. Persky and Dolwick (2020) conducted a study where they paired unpleasant and pleasant odours with VR kitchen scenes. They found that the unpleasant odour increased the sense of presence, while the pleasant scent did not have a significant effect. Additionally, providing a localized coffee scent improved participants' recall of the scent's source location. These findings suggest that scents can enhance the realism and immersion of VR experiences, but the specific effects may vary depending on the nature of the scent and the context in which it is presented.

In VR, certainly, the odour of food plays a significant role in our eating experience. The sense of smell is closely linked to our sense of taste, and together, they contribute to our overall perception of flavour (Persky and Dolwick, 2020). The aroma of food can enhance our enjoyment and appreciation of the flavours. When we chew and swallow food, volatile compounds are released from the food and enter the nasal passages, contributing to the overall sensory experience (Glezer et al., 2021). This is where during the COVID-19 when individuals infected with COVID-19 experience loss of the sense of smell (anosmia), their ability to evaluate the full flavour profile of foods is compromised (Glezer et al., 2021). This poses challenges for sensory panels responsible for assessing taste, aroma, and texture, potentially impacting the accuracy of their evaluations.

Virtual reality has the potential to greatly impact food scent identification by incorporating olfactory cues into immersive experiences (Picket and Dando, 2019). The integration of smell in VR can enhance the perception and identification of food scents, leading to a more realistic and engaging sensory experience. VR technology, combined with olfactory displays and simulations, can create a multisensory environment that enhances the users' sense of presence and provides new opportunities for education, therapy, and other applications (Picket and Dando, 2019).

VR and immersive environments have been extensively studied in the context of sensory perception and consumer behavior. Kong et al. (2020) conducted a preliminary study on the sensory perception of chocolate products in VR environments, highlighting the potential application of VR in sensory science. Crofton et al. (2019) reviewed the potential applications of VR and augmented reality technologies in sensory science, emphasizing the advancements and opportunities these technologies offer in this field. Additionally, Torrico et al. (2020) explored the effects of context and VR environments on the wine tasting experience, acceptability, and emotional responses of consumers, demonstrating the influence of VR on sensory experiences. Oliver and Hollis (2021) conducted a feasibility study on the influence of the eating environment on eating behavior using VR, indicating the potential of VR as a tool for studying environmental influences on behavior. Haar et al. (2021) utilized embodied VR for the study of real-world motor learning, demonstrating the application of VR in studying sensory-motor interactions and learning. Furthermore, Picket & Dando (2019) provided evidence of the influence of environmental immersion on hedonics, perceived appropriateness, and willingness to pay in alcoholic beverages, highlighting the impact of context, including VR, on sensory testing. The research indicates that VR and immersive environments have the potential to significantly impact sensory perception, consumer behaviour, and real-world learning, highlighting the relevance of VR in understanding the notion of scene, context, and virtualization of space in various sensory and behavioural contexts.

The aim of the study is to analyse the feasibility of odour recognition tests in VR environments. There is limited research on using VR in scent identification. This is important especially in VR when changing the food environment setting (*e.g.,* restaurant setting, open space environment such as park or beach *etc.*), it can affect the sensory cues while performing the sensory test session. Since using VR, consumer unable to see the realism of the product, food scent will be the first cue before eating.

## 2 Materials and methods

### 2.1 Study set up and technology

An empty and quiet classroom at the Hungarian University of Agriculture and Life Sciences (MATE) was dedicated to the VR experiment. The virtual sensory laboratory was implemented using Unreal Engine version 4.27.2 (Epic Games, Cary, North Carolina, US), with head-mounted displays (HMD) HTC VIVE Pro Eye (HTC Corporation, Xindian, New Taipei, Taiwan). Two student assistants had been recruited to help set up the system and instruct the participants as to what to do during the experiment.

### 2.2 Virtual environment

**Fig. 1:**
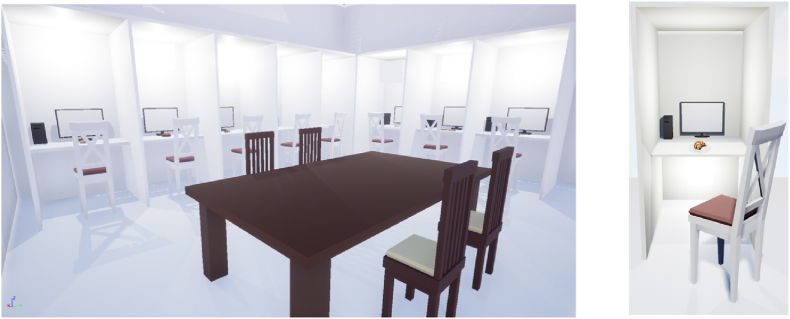
Virtual replica of MATE sensory laboratory.

The software was developed utilizing Unreal Engine version 4.27.2 by Epic Games in Cary, North Carolina, USA. The objective was to make the environment closely resemble the sensory booth at the Hungarian University of Agriculture and Life Sciences (MATE). Following the ISO 6658:2017 standard, a well-established sensory laboratory should adhere to specific criteria, including using white or light grey colours, maintaining good natural lighting at 6500 K, and ensuring proper ventilation. Within the virtual sensory laboratory, there are eight booths, each equipped with a computer, monitor, chair, and food product (see Figure 1). These booths are designed with dimensions of 1m × 1m x 2.5m. Additionally, in the centre of these booths, there is a discussion table with four chairs.

### 2.3 Measures

The participants had to answer several questionnaires and tasks from the following measures.

Pre-experiment questionnaire: Demographic information including sex, gender, age, and nationality were collected. Virtual Reality experience (*e.g.,* familiarity) were also collected for a separate investigation.

Smelling identification: Participants answer verbally, and the student assistant will record the answer.

Post-experiment questionnaire: VR Neuroscience Questionnaire which measures the quality of user experience, game mechanics, and in-game assistance, as well as the intensity of VR induced symptoms and effects (VRISE) (Kourtesis et al., 2019). Also there will be a comment section where participant put some comments on the experiment and how easy to identify the scent.

### 2.4 Scent sticks

Five (5) smelling scents were chosen: lemon, strawberry, cinnamon, vanilla, and caramel coded with three (3) digit random numbers. The chemical compound used in the scents are D-Limonene (lemon, CAS: 5989-27-5), Ethyl methylphenylglycidate (strawberry, CAS: 77-83-8), Cinnamaldehyde (cinnamon, CAS: 14371-10-9), Vanillin (vanilla, CAS: 121-33-5) and Maltol (caramel, CAS: 118-71-8). The smelling scents were inside airtight test tubes, each containing special paper strips with a concentrated form of the scents. Participants will take a whiff of these strips to experience and discover what each smell is like. The smelling test was carried out as it can be done easily on VR to determine its application within the sensory analysis. The smelling sticks’ scent of lemon, strawberry, cinnamon, and vanilla was prepared according to the ISO 5496:2006 standard.

### 2.5 Procedure

**Fig. 2:**
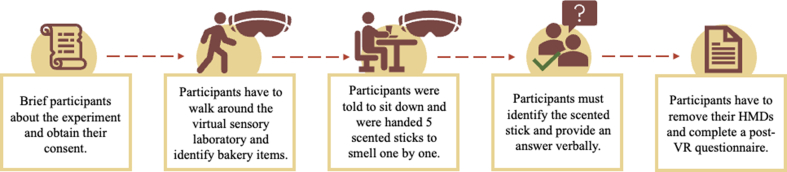
Flow of the experiment and tasks for participants.

Figure 2 shows the flow of the experiment. Participants were invited and given their consent to participate in the experiment. They were briefed about the study's expectations and aims. First, they stand in a fixed position (starting point), and then they put on the head mounted displays (HMDs) with the assistance of the student assistant (a master student as part of their project). Once the HMDs had been put on, the VR environment had been started. Five (5) virtual sensory booths were displayed in the VR environment, with each sensory booth containing a different bakery item and the participants need to complete task 1 and 2 (for further details, see 2.5.1 and 2.5.2). The participant's total time in the overall experiment is expected to range from seven (7) to ten (10) minutes. After the two tasks', the participants' HMDs were removed, and they were asked to complete the post VR questionnaire. The participant was given a candy as an incentive for participating.

#### 2.5.1 Task 1: identify the bakery items

Five (5) virtual sensory booths were displayed in the VR environment, with each sensory booth containing a different bakery item (pretzel, bread, croissant, baguette, and donut). As their first task, participants will spend at least three (3) minutes walking and identifying each product in the sensory booths. After completing the first task, participants must sit down (simultaneously in the virtual laboratory) on the chair, also with the help of the student assistant to prevent falling.

#### 2.5.2 Task 2: Smelling test

With a virtual big table and an empty plate in front of the participant, task two requires the participant to smell and identify five scented sticks (lemon, strawberry, cinnamon, vanilla, and caramel). The student assistant will hand in the test tubes containing the concentrated smelling strips one by one and asked verbally a question in the form of “What aroma do you smell?”, “Can you guess the aroma?” or “What do you think the aroma smells like?”, while the answer (verbally) will be recorded. The participant's total time in the test ranges from five (5) to seven (7) minutes.

### 2.6 Participants

The participants were students from Hungarian University of Agriculture and Life Sciences (MATE). Based on Table 1, the sample was composed of sixty (60) participants consisting of 70% female (with a mean age of 22.24 ± 4.47) and 30% male (23.67 ± 5.21). This is an adequate number of participants for acceptability test as a study from Gacula and Rutenbeck (2006), the estimated number of participants should be between 40 and 100 participants.

**Table 1:** Participants gender and age.

Gender	Number of participants (*n*)	Percentage (%)	Age				
			Mean ± SD			Min	Max
Male	18	30	23.67	±	5.21	19	39
Female	42	70	22.24	±	4.47	19	42
Total	60	100	22.67	±	4.70	19	42

Participants gave a consent, and the aim of the study was explained to consumers before the experiment started to ensure the consumer understood the methodology and that they need to use the VR headset. Ethical approval for the measurements was obtained from MATE internal ethics committee (approval number: MATE-BC/947-1/2023).

### 2.7 Data analysis

The findings were statistically interpreted and displayed in tabulated and graph form, with the mean or average value, minimum, maximum, and standard deviation. A multivariate analysis approach was applied to the result of scent identification using XLSTAT (Addinsoft, New York, USA). Multiple Correspondence Analysis (MCA) was performed on the identification of each scent whether participants are able to identify them correctly or not. Figures were prepared using XLSTAT (Addinsoft, New York, USA).

## 3 Result and discussion

### 3.1 Scent validation

Results were determined by determining whether or not each participant was able to correctly identify the five (5) scents. In sensory analyses, identifying the scent correctly without the aid of word is one of the difficult sensory analysis methods. Each scent was also examined in detail to determine which ones were similar to or closely related to the one that was chosen. These are crucial responses that may reveal participants' preferences for particular scent attributes.

### 3.2 The effect of scent and food products image on smelling experience

#### 3.2.1 Scent identification

**Fig. 3:**
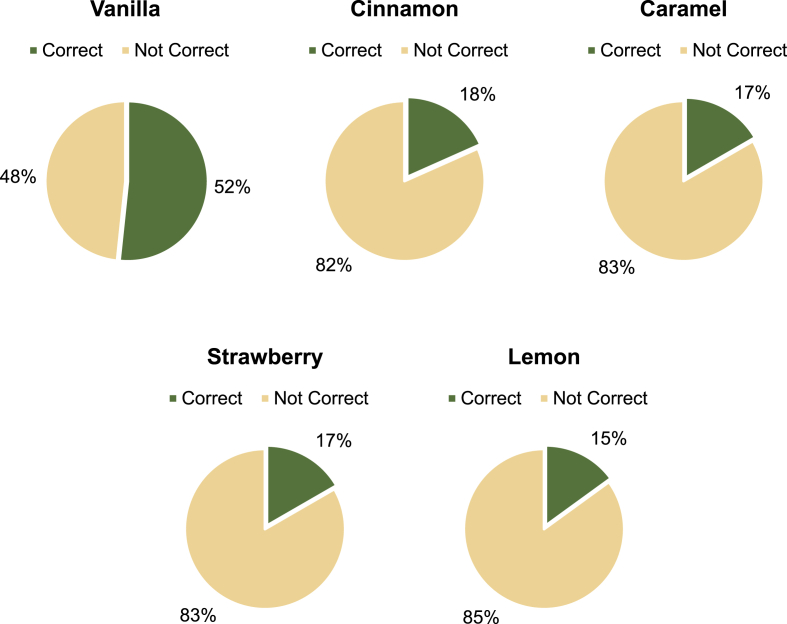
Scent identification from the participants of vanilla, cinnamon, caramel, strawberry, and lemon.

According to Figure 3, 52% of participants were successful in correctly identifying the vanilla scent. Other scents that were correctly identified but fell short of 20% included lemon (15%), strawberry (17%), cinnamon (18%), and caramel (17%). The smelling task was affected by the participants' exposure to various bakery products and manipulation of their olfactory systems in the first task of the experiment, which required them to identify various bakery items in a virtual sensory booth.

#### 3.2.2 Scent identification and within same category

**Fig. 4:**
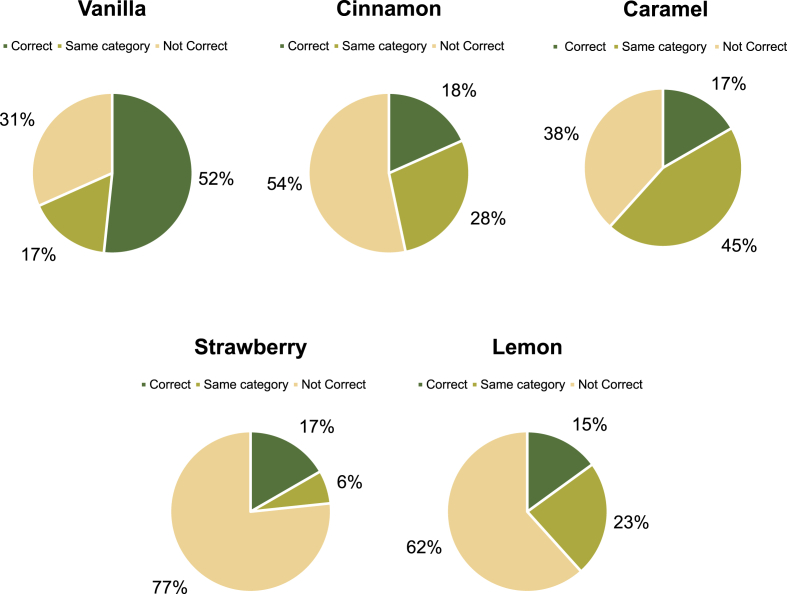
Scent identification including the same category from the participants of vanilla, cinnamon, caramel, strawberry, and lemon.

One of the easiest scents to be identify is vanilla as it is a common scent that is associated with bakery or pastry products. Figure 4 shows the detailed answer of vanilla which 52% of participants were able to identify the scent. 31% cannot identify the scent and 17% were able to identify the scent in the same category (Sweet, Sugar, Candy). The aroma of vanilla has been found to have cross modal effects on perception. In a study on cross modal correspondences between scents and shapes, vanilla was correlated with rounded shapes (Brianza et al., 2022). This suggests that the perception of vanilla scent may influence how people perceive the shape or form of bakery products, potentially enhancing the perception of softness and smoothness (Brianza et al., 2022).

The scent of cinnamon is usually associated with seasonal bakery products such as Christmas or Thanksgiving festive. Only 18% of the participants could identify the scent correctly while 28% identified on the same category (Spices, Almond). Most (54%) of the participant were unable to identify the scent but the participant had associated the smell with seasonal products. The scent of cinnamon in bakery products is often associated with feelings of nostalgia and can have a significant impact on consumer perception and behaviour (Brianza et al., 2022). The aroma of cinnamon can evoke positive emotional responses and trigger memories of past experiences, creating a sense of familiarity and comfort (Brianza et al., 2022). This nostalgic effect of cinnamon scent can contribute to the overall sensory experience of bakery products and enhance their appeal to consumers.

For the caramel scent, 38% cannot identify the scent or mixed up with vanilla scent. While 17% of the participants were able to guess the scent and 45% were guessed in the same category (Burnt, Coffee, Chocolate, Bourbon, Butter). A lot of participants guessed in the same category as caramel can be paired with smell of burnt and creamy. Caramel scent can indeed be challenging to identify in certain bakery items. The caramel scent in bakery products is a desirable and distinct aroma that adds depth and richness to various baked goods. Caramelization, which occurs when sugar is heated, plays a crucial role in the formation of the caramel scent and flavour in bakery products (Ertuğral, 2021). During the caramelization process, sugars undergo non-enzymatic chemical reactions, such as the Maillard reaction and caramelization, resulting in the formation of various aroma compounds (Ertuğral, 2021).

77% of the participants were unable to identify the strawberry scent while 17% were able to identify correctly. The other 6% can identify on the same category (Berry, Raspberry). This is a bit difficult to identify as the bakery items shown does not related with strawberry scent. While strawberry is considered to have a distinct and recognizable aroma, it may not always be easy to identify in bakery products due to the presence of other ingredients and flavours (Choudhary et al., 2021). Research has shown that the aroma of strawberry is complex and consists of various volatile organic compounds that contribute to its characteristic scent. These compounds work together to create the unique and fruity aroma of strawberry (Szakál et al., 2022).

The scent of lemon was the least scent that can be identify among all other scents. Only 15% of participants can identify the scent while 23% able to identify in the same category (Citrus, Orange, Vitamin C). While 62% of participants cannot identify the scent of lemon. Identifying the lemon scent in bakery products can be challenging due to the complex nature of the aroma and the presence of other ingredients and flavours. Lemon essential oil is commonly used as a flavouring agent in bakery products, including confectionery, desserts, and baked goods, due to its characteristic aroma profile (Incegul et al., 2018). While these components can contribute to the flavour profile of the baked goods, the scent might not be as prominent as in other contexts like cleaning products or personal care items (Incegul et al., 2018).

Scents and VR were used in Brengman et al. (2022) and Flavián et al. (2021) studies. Both the smell and the exposure were affected by the environment or image's set off. The perception of food smell and the influence of the environment or image's set off in VR can be understood through the concept of cross-modal correspondences and the impact of sensory cues on perception. Odour quality and the ability to discriminate odours can be affected by previous experiences and associations (Adams et al., 2014). This suggests that the environment or image's set off in VR, which includes visual cues, can influence the perception of food smell by activating relevant memories and associations.

### 3.3 Analysis of smell identification

**Fig. 5:**
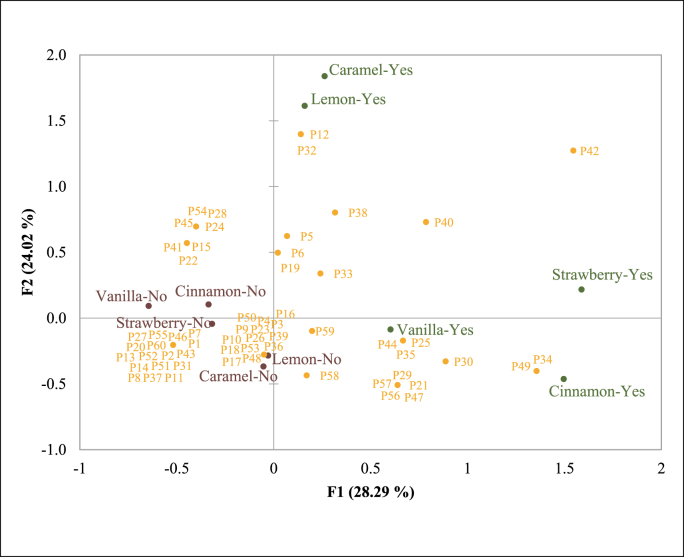
Multiple Factor Analysis (MFA) on the participants who can and cannot identify the scent (vanilla, cinnamon, caramel, strawberry, and lemon). YES and NO indicates if the scent was identified correctly or not. Scents closer to each other indicate that they were identified by the same participants.

Based on Fig. 5, only one participant (P42) was able to identify all the scent while 17 participants were unable to identify any of the scents. The position of lemon and caramel (yes) is closely related, same goes with lemon and caramel (no). While vanilla, cinnamon, and strawberry (yes) are closely related with each other and also vanilla, cinnamon and strawberry (no). Vanilla was identified by the most participants, which is expected as vanilla has been shown as one of the most pleasant (liked) odour (Arshamian et al., 2022). These results are interesting as for example that lemon and caramel there are a scent note that participant can identify correctly or incorrectly.

These can be validated further by some participants given a positive and informative comments throughout the experiment. As all the participants do not have experience with the VR before, most comments “It was a very interesting experience” and “It was a very good experience”. There are also some interesting comments that the image from the virtual sensory laboratory had an effect with the stick that they are smelling, “It was a thrilling experience, and it was interesting how our senses (primarily sight) can be deceived”, “It was surprisingly easy to move around in the virtual space, it was very lifelike, recognizing scents was not easy” and “I felt the pictures made me smell different than what it actually was”.

## 4 Conclusion

Virtual reality environments are generally applicable for conducting odour recognition tests. VR has an advantage that visual cues can be easily manipulated and changed compared to the odours presented to the participants.

Presence, perception, and user experience are crucial factors in determining the effectiveness of VR environments. While visual and auditory stimuli have been extensively studied, the role of scents in VR environments has received less attention. The environment or image's set off in VR refers to the specific context or visual stimuli presented to the user, which can influence their sensory perception and response. Presence refers to the subjective feeling of “being there" in a virtual environment. Scent, as a powerful sensory cue, can enhance the realism and immersion of the virtual environment. Perception, on the other hand, refers to the interpretation and understanding of sensory information. Scent can influence perception by eliciting emotional and cognitive responses.

In conclusion, scent can have a significant impact on presence, perception, and user experience in VR environments. Overall, the research suggests that scent can enhance presence, improve recall, influence user comfort and emotions, and shape consumers' reactions and behaviour in VR environments. However, it is important to note that the effects of scent can vary depending on factors such as the type of scent, the context, and individual preferences. Further research is needed to better understand the underlying mechanisms and optimize the use of scent in VR experiences.

## CRediT authorship contribution statement

**Abdul Hannan Bin Zulkarnain:** Conceptualization, Methodology, Software, Formal analysis, Investigation, Writing – original draft, Writing – review & editing, Visualization. **Dalma Radványi:** Writing – review & editing. **Dorina Szakál:** Writing – original draft, Writing – review & editing. **Zoltán Kókai:** Supervision, Writing – original draft, Writing – review & editing. **Attila Gere:** Conceptualization, Methodology, Validation, Writing – original draft, Writing – review & editing, Supervision.

## Declaration of competing interest

The authors declare that they have no known competing financial interests or personal relationships that could have appeared to influence the work reported in this paper.

## Data Availability

Data will be made available on request.
